# Supplementary material for: Dynalign II: common secondary structure prediction for RNA homologs with domain insertions
Source: Nucleic Acids Res. 2014 Nov 21;42(22):13939–48. doi: 10.1093/nar/gku1172 (PMC4267632; doi:10.1093/nar/gku1172)
Supplement: SUPPLEMENTARY DATA [file supp_gku1172_nar-02021-z-2014-File012.zip › manual/GUI/html/Sequence_Editor.html]

RNAstructure GUI Help -- Sequence Editor


|  |  |  |
| --- | --- | --- |
|  | RNAstructure GUI Help Sequence Editor | - Contents - Index |
| **How to Create and Edit an RNA or DNA Sequence**  For a new sequence, select "File -> New Sequence" from the menu. This brings up an empty sequence editor window. Type a title into the title box, if desired. Then, click the comment box with your mouse. Here, you may type a comment of any number of lines, if you wish. Finally, move to the sequence box and type in your sequence 5' to 3'.  You may also use X to represent a gap or unknown base. Before exiting, select "File -> Save Sequence" or "File -> Save Sequence As..." to save the sequence. All sequences are saved in the SEQ format.  WARNING: Type in most of the sequence with CAPITAL letters. When sequences are folded, any **lowercase bases are not allowed to base pair**. You may use T's, U's, or both. The RNA folding algorithm assumes the sequence is RNA and will interpret a T as a U. The DNA folding module will likewise assume DNA and interpret U as T.   - ```   Right: ACCGGAUUCAGAUAG   ``` - ```   Wrong: accggauucagauag   ```  ---  **Loading Sequences**  To load a sequence, select "File -> Open Sequence." Three types of files can be loaded: sequence (SEQ) files, Genbank files, and simple text files. Sequence files follow a specific sequence format (See "Appendix A: File Formats"), Genbank files follow the Genbank format, and simple text files contain a sequence of bases with nothing else.   ---  **Editor Features**  For ease of entering a sequence, the RNAstructure editor can give audio feedback as a sequence is entered. If your computer has sound capabilities, there are two different ways by which a structure can be read out loud. If the "Read While Typing" checkbox under the "Read" menu is selected, bases will be read out loud as they are typed into the editor. and the "Read While Typing" checkbox is selected, you will hear the sequence being read as it is typed into the editor. If the "Read Sequence" button is pressed, the sequence will be read from the beginning in its entirety. The "Stop Reading" button will stop this automatic reading at any time. When the "Read Sequence" button is pressed again, reading restarts at the beginning of the sequence.  The editor can format a sequence into a Genbank-like format. Pressing the "Format" button will divide a sequence into lines of 50 bases, with spaces after every 5.  The "Fold RNA" and "Fold DNA" buttons will input the sequence directly into the "Fold" module, with the appropriate nucleic acid type selected. RNAstructure will request that the sequence be saved as a sequence file first, if the sequence has not been saved. | | |
| Visit The Mathews Lab RNAstructure Page for updates and latest information. | | |
